# Novel Pathophysiological, Diagnostic and Therapeutic Concepts in Acute and Recurrent Pericarditis

**DOI:** 10.31083/j.rcm2403077

**Published:** 2023-03-03

**Authors:** Aldo Bonaventura, Georgia K Thomas, Michele Golino, Adolfo Gabriele Mauro, Alessandra Vecchié, Marco Giuseppe Del Buono, Stefano Toldo, Nicola Potere, Antonio Abbate

**Affiliations:** ^1^S.C. Medicina Generale 1, Medical Center, Ospedale di Circolo e Fondazione Macchi, ASST Sette Laghi, 21100 Varese, Italy; ^2^VCU Pauley Heart Center, Virginia Commonwealth University, Richmond, VA 23220, USA; ^3^Department of Medicine and Surgery, University of Insubria, 21100 Varese, Italy; ^4^Department of Cardiovascular Medicine, Fondazione Policlinico Universitario A. Gemelli IRCCS, 00161 Rome, Italy; ^5^Department of Cardiovascular and Pulmonary Sciences, Catholic University of the Sacred Heart, 00161 Rome, Italy; ^6^Department of Medicine, Division of Cardiovascular Medicine and Robert M. Berne Cardiovascular Research Center, School of Medicine, University of Virginia, Charlottesville, VA 22908, USA; ^7^Department of Innovative Technologies in Medicine and Dentistry, “G. D'Annunzio" University, 66100 Chieti, Italy

**Keywords:** acute pericarditis, recurrent pericarditis, NLRP3 inflammasome, IL-1α, IL-1β, anakinra, rilonacept, inflammation

## Abstract

Acute pericarditis is the most frequent pericardial disease characterized by inflammation of the pericardial layers resulting in pain, dyspnea and fatigue. Often limited to an isolated event, up to 30% of patients experience one or more recurrences. There is limited knowledge about the pathophysiology of this disease, possibly due to the limited availability of animal models. More recently, following seminal clinical trials with colchicine and interleukin-1 (IL-1) blockers and a novel murine model of acute pericarditis using zymosan A, it has become clear that the NLRP3 (NACHT, leucine-rich repeat, and pyrin domain-containing protein 3) inflammasome/IL-1β axis plays a central role in driving acute pericardial inflammation and in sustaining this process during recurrences. Diagnostic management of pericarditis has been implemented with multimodality imaging including echocardiography, cardiac computed tomography, and cardiac magnetic resonance. These imaging modalities provide essential diagnostic and pathogenetic information, and are able to characterize pericardial inflammation, allowing to refine risk stratification and personalize treatment. Recent acquisitions yield relevant implications with regard to the therapeutic management of acute and recurrent pericarditis. Non-steroidal anti-inflammatory drugs (NSAIDs) and colchicine are cornerstone therapies either for acute and recurrent pericarditis. However, the benefits of targeted agents, such as anakinra — a recombinant human IL-1 receptor antagonist — and rilonacept — an IL-1α/IL-1β trap, are being increasingly recognized. To this end, phenotyping patients with pericarditis and addressing such therapies to those presenting with auto-inflammatory features (elevated C-reactive protein, sustained pericardial and systemic inflammation, multiple recurrences) is of utmost importance to identify patients who might be more likely to benefit from NLRP3 inflammasome/IL-1β pathway blockade.

## 1. Introduction

Acute pericarditis is the most frequent pericardial disease, and an increasingly recognized cause of chest pain, with an estimated incidence of 27.7 cases per 100,000 persons/year [1]. As a complication of acute pericarditis, recurrences may occur in up to 30% of cases within 18 months after a first episode, especially among patients not treated with colchicine [2, 3, 4].

The latest 2015 European Society of Cardiology (ESC) guidelines recommend non-steroidal anti-inflammatory drugs (NSAIDs) or aspirin and colchicine as an initial treatment either for the first episode or for recurrences [5]. Recently, essential steps have been accomplished to enlighten the pathophysiology and therapy for acute and recurrent pericarditis. Specifically, a focus has been placed on the emerging role of both NACHT, leucine-rich repeat, and pyrin domain-containing protein 3 (NLRP3) inflammasome and interleukin-1β (IL-1β) in driving the onset of the acute pericardial inflammation, yet further sustaining the inflammatory process during recurrences [6, 7, 8]. In addition, the RHAPSODY (Rilonacept inHibition of interleukin-1 Alpha and beta for recurrent Pericarditis: a pivotal Symptomatology and Outcomes stuDY) trial with rilonacept — an IL-1α and IL-1β trap — has shown that IL-1 blockade is able not only to resolve the acute flare of pericarditis rapidly but also to decrease the risk of recurrences [9].

This review summarizes recent evidence about pathophysiology, diagnosis, and therapy in acute and recurrent pericarditis. The advancements in this field appear of utmost importance, especially in managing patients experiencing recurrent episodes, as a specific treatment, i.e., rilonacept, is now approved for treating this condition [10].

## 2. Novel Pathophysiological Clues: A Central Role for the NLRP3 Inflammasome/IL-1β Axis

**Fig. 1.**
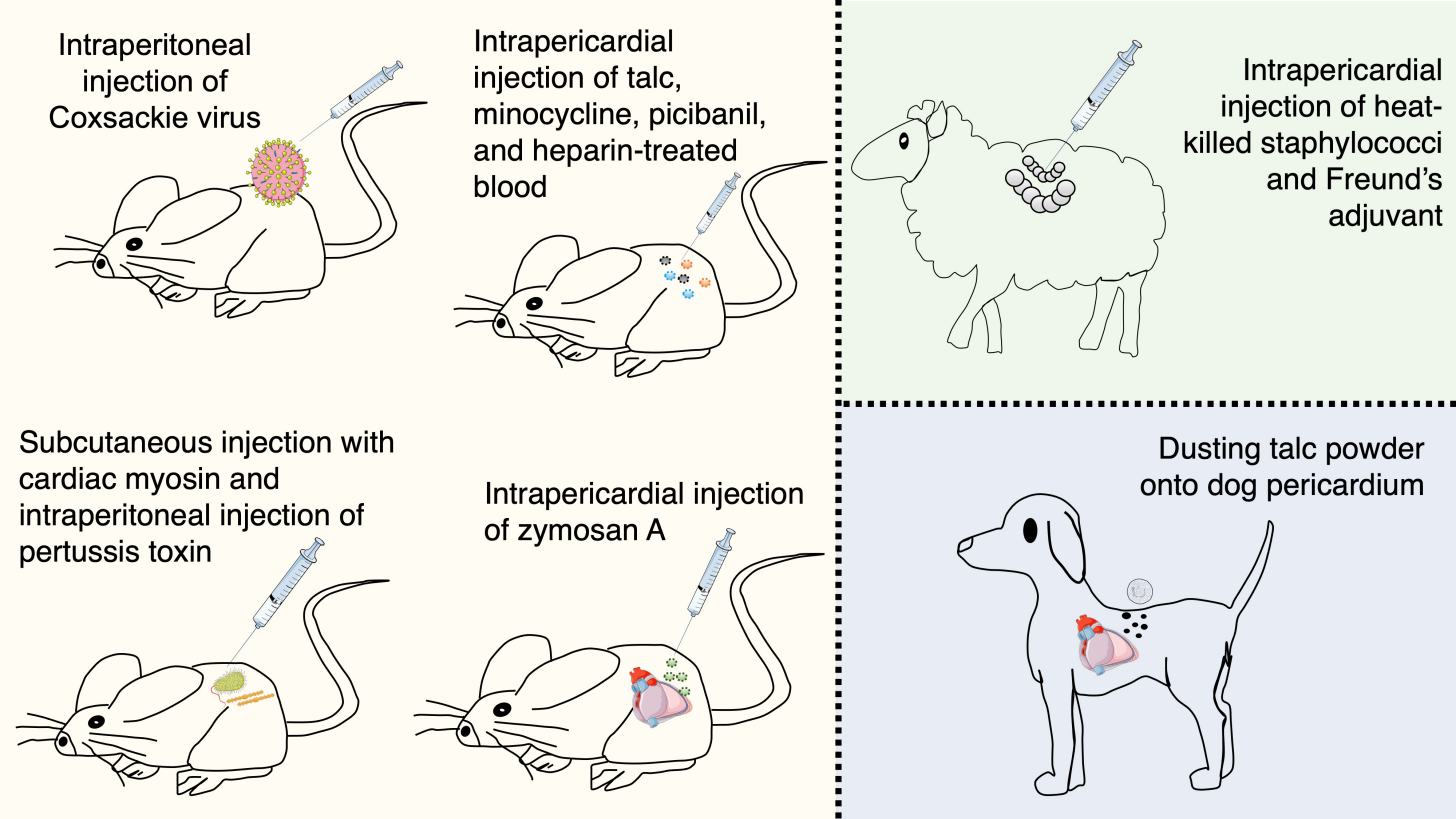
** Animal models that investigated acute pericarditis**. A limited number of animal models has been available for many years, thus explaining the limited knowledge about pericarditis pathophysiology and the availability of very few therapies. Interestingly, most of the stimuli of these animal models had a direct, yet previously unknown, link with the NLRP3 inflammasome. Indeed, hydrated magnesium silicate (talc) and Freund’s adjuvant containing aluminum are canonical stimuli for the NLRP3 inflammasome activation. An innovative murine model of acute pericarditis is based on intrapericardial injection of zymosan A that generates an inflammatory reaction of the pericardium.

For many years, acute pericarditis has been thought to be initiated by a virus, including the more recently emerged severe acute respiratory syndrome coronavirus 2 (SARS-CoV-2) [11]. However, when a precise etiology cannot be determined, it is usually considered to be “idiopathic” [12]. The small number of animal models and pathology studies may have accounted for a long time for a scarce understanding of the disease and the consequential absence of targeted therapies (Fig. 1).

The first available evidence of an animal model of pericarditis dates back to 1980 when Matsumori *et al*. [13] proposed a mouse model of chronic perimyocarditis obtained through intraperitoneal injection of Coxsackie virus B3 (Table 1, Ref. [6, 13, 14, 15, 16, 17]). In this model, as the virus stimulated chronic inflammation, a buildup of inflammatory cells — histiocytes, lymphocytes, plasma cells, and a few polymorphonuclear leukocytes — was evident starting from day 14 with perimyocardial fibrosis appearing at day 28 [13]. In 1986 Pagé *et al*. [14] reproduced the features of acute pericarditis in animals by dusting talc powder on dogs’ pericardium with a gauze (Table 1). A year later, Leak *et al*. [15] further recreated acute pericarditis in sheep through intrapericardial injection of heat-killed staphylococci in addition to Freund’s adjuvant (Table 1). The authors established an exceptionally complete animal model, able to recapitulate step-by-step all stages of pericardial inflammation up to a complete resolution of the inflammatory process [15]. The early phases were indeed characterized by neutrophil infiltration, edema, and accumulation of flocculent material in the submesothelial space. This process became even more organized at day 7, with an abundance of neutrophils, macrophages, erythrocytes, and fibrin deposition. At this stage, denudation of the mesothelial lining was observed, which favored platelet adherence and accumulation of neutrophils and macrophages. A localized healing process was observed starting from the second week, which ended after nearly nine months when the pericardial layers recovered entirely. Alternatively, Afanasyeva *et al*. [16] proposed a mouse model of perimyocarditis leading to pericardial constriction through subcutaneous injection with cardiac myosin emulsified in complete Freund’s adjuvant and intraperitoneal injection of pertussis toxin (Table 1). This model was specifically created to evaluate the clinical consequences of constrictive pericarditis. Kojima *et al*. [17] generated their mouse model by mimicking the post-operative pericardial adhesions (Table 1). To achieve this, they tested low- and high-doses of talc, minocycline, picibanil (lyophilized mixture of group A *Streptococcus pyogenes*), and heparin-treated blood. Finally, they found that talc was indeed the agent able to induce pericardial adhesions to the greatest extent, confirming what was previously described by Pagé *et al*. [14].

**Table 1.** **Animal models of acute and chronic pericarditis**.

Authors	Animal	Condition	Technique	Major findings
Matsumori and Kawai [13]	Mouse	Chronic perimyocarditis	Intrapericardial injection of Coxsackie virus B3 (0.1 mL of virus suspension)	One-third of mice died after virus injection.
				Initial histopathological changes occurred mainly in myocardial fibers with a limited amount of inflammatory cells, that increased by day 14.
				On day 28, perimyocardial fibrosis was more evident while cellular infiltration gradually decreased. Perimyocardial fibrosis was marked over days 90 to 180.
Pagé *et al*. [14]	Dog	Acute pericarditis	After pericardiotomy, atrial surfaces were dusted with sterile talcum powder. No information about the quantity of the talcum powder was provided.	No details about histopathological alterations were discussed.
Leak *et al*. [15]	Sheep	Acute pericarditis	The pericardial sac was exposed through left thoracotomy at the 5th intercostal space. The pericardial cavity was inoculated with bacterial toxin (0.2 g of dried bacterial cells from* Staphylococcus aureus*), complete Freund’s adjuvant (3 mL), and sterile phosphate-buffered saline (3 mL) under sterile conditions. The intrapericardial injection was suspended in a syringe with a 21-gauge needle to create a smooth emulsion. Then, 5 mL of the emulsion were injected into the pericardial cavity.	∙ 3 to 24 h: Changes in the shape of the mesothelial cells that appeared rounded, as if contracted, were evident as well as initial neutrophil infiltration, edema, and accumulation of flocculent material in the submesothelial space already after 3 h.
				∙ 6 to 24 h: Accumulation of a brownish sero-sanguinous fluid containing a large number of neutrophils, macrophages, red blood cells, and fibrin strands was recorded. It was also evident pericapillary edema, extravasation of red blood cells and neutrophils, and swelling of capillary endothelial cells and myocytes from the underlying myocardium. Denudation of the mesothelial lining, favoring the adherence of platelets, neutrophils, and macrophages, was also observed.
				∙ 48 to 72 h: A large amount of fibrin was observed on the visceral and parietal surfaces of the pericardium as well as a large number of inflammatory cells. The mesothelium was detached as a result of the inflammatory injury.
				∙ 6 to 8 days: Many neutrophils and macrophages were observed as aggregates included in a filamentous network, while fibrin strands were progressively broken down.
				∙ 2 weeks: A process of local healing was recorded, with a large increase in the amount of connective tissue containing fibroblasts, lymphocyte aggregates, while the number of neutrophils decreased.
				∙ 9 months: A complete recovery of the pericardial surfaces was achieved with evidence of small infiltrates of lymphocytes and fibroblasts surrounded by bundles of collagen and elastic fibers.
Afanasyeva *et al*. [16]	Mouse	Perimyocarditis evolving toward constriction	Subcutaneous injections of 200 to 250 g of cardiac myosin emulsified in complete Freund’s adjuvant on days 0 and 7, and intraperitoneal injection of 500 ng of pertussis toxin on day 0.	Cardiac myosin–induced experimental autoimmune myocarditis in IFN-γ–KO mice developed pericarditis leading to a constrictive phenotype. The pericardium in KO mice was thickened with white discoloration causing adhesions between the two pericardial layers and to the pleura, diaphragm, and chest wall.
				The cellular infiltrate of the pericardium included poly- and mononuclear cells including eosinophils. Mesothelial hyperplasia and mesothelial reaction (i.e., change to cuboidal morphology of mesothelial cells, typical of pericardial injury) was also described.
Kojima *et al*. [17]	Mouse	Acute post-operative pericarditis	Following skin incision in the abdomen and cut of the peritoneum to reach the abdominal cavity, an intrapericardial injection of 500 μL of low- (2.5 mg/g) or high-dose talc (5 mg/g), 300 μL of minocycline (2 mg/mL), 375 μL of picibanil (lyophilized mixture of group A Streptococcus pyogenes; 3.0 KE/kg), 300 μL of heparin-treated blood from donor mice, or saline solution was performed from the diaphragm side through a 23-gauge needle from a 1 mL syringe.	Only talc-injected mice showed diffuse and marked pericardial adhesions over the whole heart within 2 weeks visible.
				A large amount of macrophages and myofibroblasts, together with elastic fibers and myocardial erosion, were found.
Mauro, Bonaventura *et al*. [6]	Mouse	Acute pericarditis	After an incision on the left part of the thorax in the region of the 3rd–4th and fourth rib, muscle layers were dissected to expose the interosseous space and access the thoracic cavity. By carefully lifting the pericardial sac, zymosan A (1 mg dissolved in 50 μL of sterile NaCl 0.9%) was injected into the pericardial space througha 30-gauge needle until a complete distribution of the solution into the pericardium was observed.	Seven days after the intrapericardial injection of zymosan A, mice developed typical stigma of local inflammation:
				∙ A significantly larger pericardial effusion (+83%) was evident compared with sham (*p *< 0.001). This was already there at day 3.
				∙ A significant increase (+45%) in the visceral pericardial thickness compared with sham-operated mice (*p* = 0.016) was observed through a morphometrical analysis on hematoxylin and eosin–stained heart sections. No fibrinous deposits were found.
			After surgery, mice were randomly treated to the following pharmacological agents through intraperitoneal injection (over a period of 1 week): ibuprofen (100 mg/kg/day), colchicine (100 μg/kg/day); 3) 16673-34-0, an NLRP3 inflammasome inhibitor (100 mg/kg/day), anakinra (100 mg/kg twice daily), recombinant murine IL-1 trap, (1, 5, and 30 mg/kg/day every 48 h), and matching volume of vehicle (NaCl 0.9%). All drugs were administered after surgery and then once daily with the exception of anakinra, given twice daily, and IL-1 trap, given once every 48 h.	∙ Mice treated with zymosan A showed a 60-fold increase expression of ASC compared with sham mice meaning activation of the NLRP3 inflammasome (*p *< 0.001).
				∙ At day 3, a larger expression of IL-α and IL1-β was found both at a transcriptional and translational level in mice treated with zymosan A compared with sham-operated mice.
				∙ No impairments in cardiac function were observed in mice injected with zymosan A and in sham mice neither at 3 nor at 7 days.
				Pharmacological treatments:
				∙ Ibuprofen reduced pericardial effusion compared with vehicle-treated mice by 42% (*p *< 0.001).
			Transthoracic echocardiography was performed at day 3 and 7 to measure the amount of pericardial effusion. At day 7, mice were sacrificed and hearts harvested to get the pericardium for hematoxylin and eosin staining to measure pericardial thickness and immunofluorescence and immunohistochemistry stainings to look for the 3 components of the NLRP3 inflammasome (the sensor protein NLRP3, the scaffold protein ASC, and the effector protein caspase-1).	∙ Colchicine and the NLRP3 inh 16673-34-0 significantly reduced pericardial effusion at day 7 by 28% and 46%, respectively (*p *< 0.010 for both). NLRP3 inhibition with 16673-34-0 significantly reduced pericardial thickening by 32% (*p* = 0.003) Both colchicine and the selective NLRP3 inh reduced ASC aggregation (–93% and –78% vs. vehicle-treated mice, respectively, *p *< 0.001).
				∙ Anakinra decreased pericardial effusion by 13% compared with the vehicle group (*p *< 0.050) and the same did IL-1 trap given every 48 h (–43%, –35%, and –33% at all 3 doses tested, respectively, vs. vehicle-treated group; *p *< 0.010 for all). Anakinra reduced pericardial thickening by 20% (*p *< 0.050 vs. vehicle), while IL-1 trap was even more effective (–36%, –42%, and –44%, respectively, *p *< 0.001 for all). Finally, inflammasome formation, as indicated by ASC aggregates, was significantly reduced by anakinra (–75% vs. vehicle, *p *< 0.001) as well as by all doses of IL-1 trap (–85% for 1 mg/kg, –69% for 5 mg/kg, and –96% for 30 mg/kg, *p *< 0.001 for all).

Legend. IFN, interferon; KO, knockout; NLRP3, NACHT, leucine-rich repeat, and pyrin domain-containing protein 3; NLRP3 inh, NLRP3 inhibitor.

**Fig. 2.**
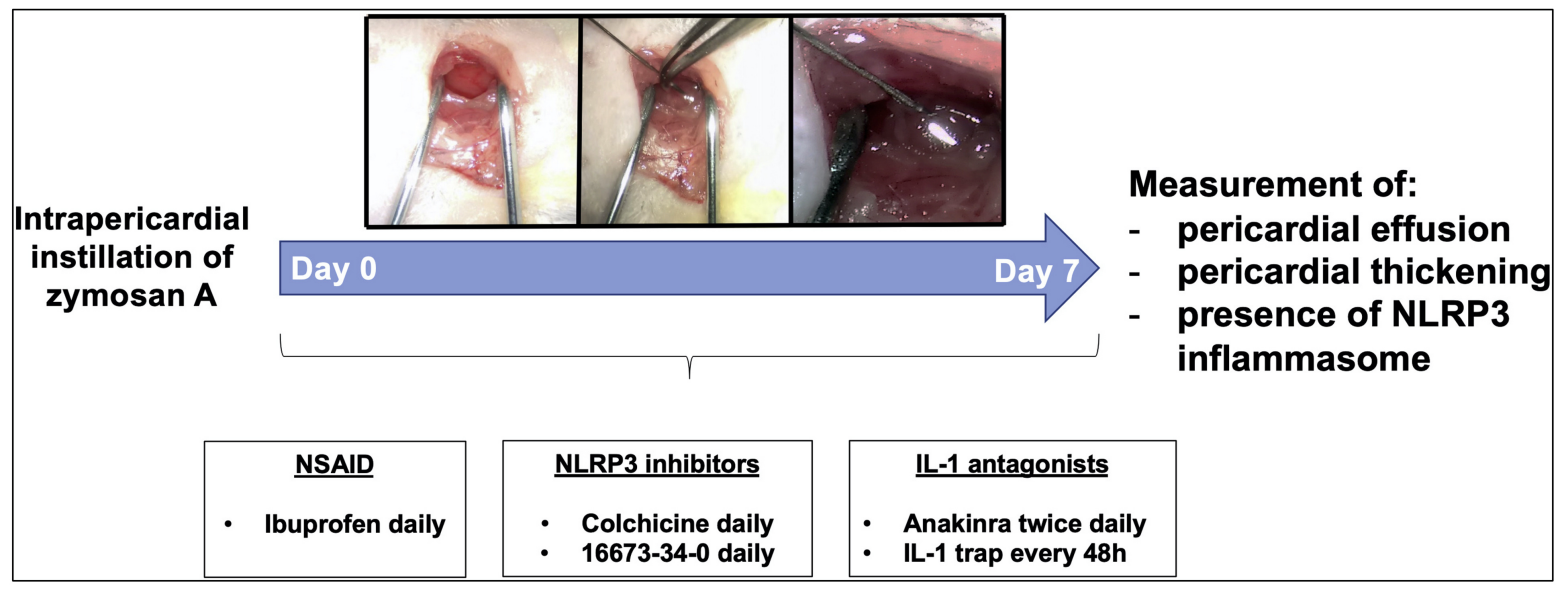
**A novel murine model of acute pericarditis induced by zymosan A**. Injection of zymosan A into the pericardial sac induces a local inflammatory reaction. Following intrapericardial zymosan A injection, augmented pericardial effusion and thickness were observed in parallel with NLRP3 inflammasome activation. Reproduced with permission from “The Role of NLRP3 Inflammasome in Pericarditis: Potential for Therapeutic Approaches”, Mauro AG and Bonaventura A* et al*., JACC Basic Transl Sci. 2021 Feb 22; 6 (2): 137–150 [6].

Although previously unknown, most of the stimuli used in the previously described animal models of pericarditis have a direct link to the NLRP3 inflammasome. Indeed, hydrated magnesium silicate — talc — was shown to induce NLRP3 inflammasome activation [18]. Similarly, in the model by Leak *et al*. [15], bacterial products of the Freund’s adjuvant, which also contains aluminum, are considered canonical stimuli for the NLRP3 inflammasome activation [19]. This evidence suggests an inflammasome-dependent model of acute pericarditis. A novel murine model of acute pericarditis secondary to NLRP3 inflammasome activation has been recently developed [6] (Table 1). This mouse model is based on intrapericardial injection of zymosan A that generates a local inflammatory reaction (Fig. 2, Ref. [6]). This technique shares similarities to what was previously done in animal models to cause peritonitis or arthritis [20, 21]. Zymosan A is a cell wall extract derived from the yeast *Saccharomyces cerevisiae* and an agonist of the toll-like receptor-2 that activates the NLRP3 inflammasome [22, 23]. As expression of pericardial inflammation, we were able to observe the following manifestations: pericardial effusion (83% increase at the time of sacrifice compared to sham mice), pericardial thickness (45% increase compared to sham), and ASC (apoptosis-associated speck-like protein containing a COOH-terminus caspase activation and recruitment domain) expression (a 60-fold increase compared to sham) [6]. ASC represents the scaffold for NLRP3 inflammasome assembly [24], hence increased expression of ASC through formation of dense areas of aggregation — termed specks — is indicative of NLRP3 inflammasome oligomerization [25, 26]. In addition, pharmacological blockade of the NLRP3 inflammasome improved pericardial inflammation. Mice were treated with ibuprofen (an NSAID), colchicine (an indirect blocker of the NLRP3 inflammasome), 16673-34-0 (an experimental NLRP3 inflammasome inhibitor) [27], anakinra (a recombinant human IL-1 receptor antagonist), and a recombinant murine IL-1 trap (able to bind and block IL-1α and IL-1β, an equivalent for rilonacept in humans) (Fig. 2). Although all pharmacological agents tested could alleviate inflammation to some extent, drugs directly or indirectly targeting the NLRP3 inflammasome pathway (i.e., colchicine, 16673-34-0, anakinra, and IL-1 trap) were able to attenuate the pathological changes occurring after acute pericarditis. The IL-1 trap decreased ASC expression as well as pericardial effusion and thickness in a dose-dependent fashion [6].

Although large animals (sheep or dogs) might better recapitulate the pathogenetic mechanisms occurring in human pericarditis and be easier to work with from a practical standpoint (Fig. 1), they also carry numerous disadvantages, such as higher housing and handling costs. On the contrary, using a mouse model may allow a more straightforward and wider replication at limited costs. Additionally, using genetically modified mice may enable the study of alternative molecular pathways involved in the pathophysiology of pericarditis.

The innovative murine model strongly supports a pivotal role of the NLRP3 inflammasome/IL-1β axis in the pathophysiology of acute and recurrent pericarditis. Pre-clinical findings also corroborated the positive results obtained by the RHAPSODY trial [9]. This evidence may pave the way to further in-depth mechanistic studies investigating molecular and cellular pathways of both innate and adaptive immunity in the pathophysiology of acute pericarditis to improve the treatment of this condition [28]. The accumulating evidence points to a role for the NLRP3 inflammasome/IL-1β axis in the auto-inflammatory process sustaining recurrent pericarditis, as outlined by a recent work [29].

## 3. Diagnosis: An Increasingly Large Armamentarium

According to 2015 ESC guidelines, pericarditis is classified as acute, incessant, recurrent, or chronic [5]. Acute pericarditis is diagnosed in the presence of at least two out of the following four criteria: (i) chest pain; (ii) pericardial rubs; (iii) electrocardiogram (ECG) changes; (iv) new or worsening pericardial effusion [5]. Sharp chest pain with rapid onset, typically worsened by inspiration or coughing, and alleviated by leaning forward or sitting up, is characteristic of acute pericarditis. Additional manifestations may include a dull or throbbing chest pain radiating to the trapezius ridge, low-grade fever or sinus tachycardia, which may be accompanied by non-cardiac manifestations (e.g., arthritis, rash, weight loss, night sweats) when pericarditis is associated with a systemic disease [30]. Regarding ECG changes, PR segment depression is rather sensitive and specific for pericarditis, along with diffuse ST-segment elevation. However, PR segment depression may often be the only ECG modification, while nondiagnostic or atypical changes are found in up to 40% of patients [30]. More than 30% of patients with pericarditis exhibit elevation in serum troponin I or T, or signs of myocardial involvement without new-onset abnormalities in left ventricular function upon imaging. Inflammatory biomarkers such as erythrocyte sedimentation rate (ESR), white blood cells (WBC), and C-reactive protein (CRP) are increased in approximately 80% of patients having acute pericarditis, however overall sensitivity and specificity are low. Acute pericarditis can progress to recurrent pericarditis in up to 30% of cases [3, 4]. Recurrent pericarditis is defined in the presence of signs and symptoms of acute pericarditis after a symptom-free window of at least 4–6 weeks after a prior episode of pericarditis [5, 31].

**Fig. 3.**
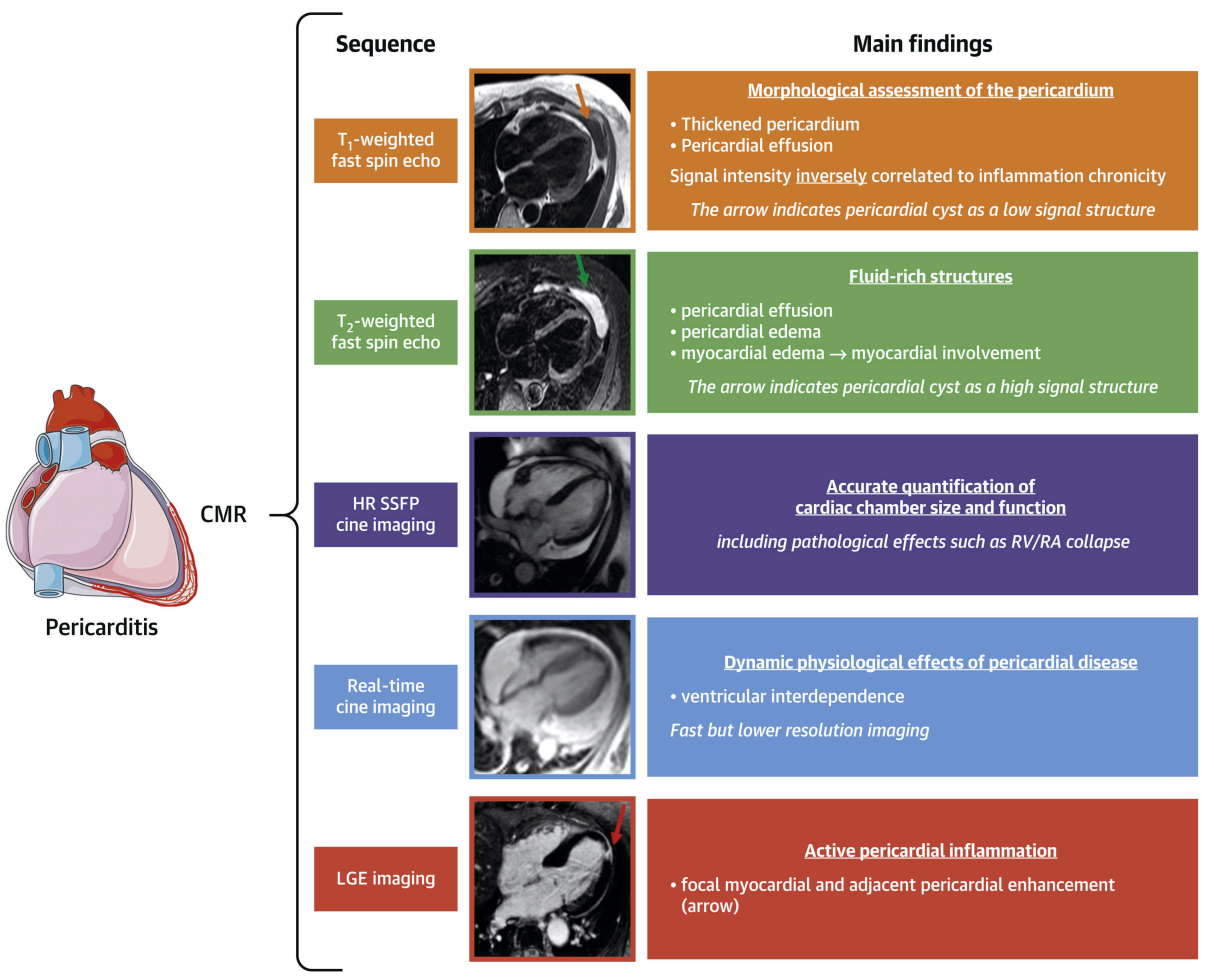
**Main findings at CMR in patients with acute pericarditis**. Reproduced with permission from “Management of Acute and Recurrent Pericarditis: JACC State-of-the-Art Review”, Chiabrando JG and Bonaventura A *et al*., J Am Coll Cardiol. 2020 Jan 7; 75 (1): 76–92 [30].

Echocardiography, cardiac computed tomography (CCT), and cardiac magnetic resonance (CMR) are the most commonly used imaging techniques to assess and characterize pericardial pathology and associated myocardial involvement [32, 33]. Major findings at cardiac multimodality imaging and their clinical relevance in patients with pericarditis are reviewed in detail elsewhere [34]. Echocardiography is considered a first-line imaging test, whereas CCT and CMR are generally used in case of inadequate echocardiographic images or diagnostic uncertainty and/or to determine the severity of illness. Although normal in about 40% of cases, transthoracic echocardiography is essential to evaluate ventricular dysfunction or possible complications (e.g., constrictive pericarditis, cardiac tamponade), to quantify pericardial effusion, and to monitor response to medical treatments. CCT provides morphological information, being the most accurate for the measurement of pericardial thickness and the most sensitive in identifying pericardial calcifications [34]. While CCT is not primarily suggested for the diagnosis of cardiac tamponade, it may be more informative to investigate constrictive pericarditis. CCT requires iodinated contrast, exposes patients to ionizing radiations, and provides minimal hemodynamic information, which limit its clinical usefulness and make it unsuitable for serial evaluations [34]. CMR has emerged as the most comprehensive imaging technique to interrogate the pericardium and adjacent myocardium as it offers both morphological and hemodynamic information by integrating several sequences within the same study [34, 35]. Late gadolinium enhancement (LGE) provides accurate information regarding the presence and degree of pericardial inflammation with very high sensitivity, and it is positively associated with histological inflammatory and neovascularization markers [36] (Fig. 3, Ref. [30]). Patients with multiple recurrences and LGE achieve significantly lower clinical remission rates [37]. Pericardial thickening at CMR and CRP elevation have been shown to predict the occurrence of adverse events, while the presence of LGE conferred lower risk [38]. In addition, in patients with recurrent pericarditis, CMR-guided management was associated with lower recurrence and pericardiocentesis rates together with decreased use of glucocorticoids [39]. Pericardial inflammation, as evaluated through LGE measurement combined with the assessment of pericardial edema in $T_{2}$-weighted sequences, may provide additional information since marked LGE with augmented signal in $T_{2}$-weighted sequences is suggestive of acute inflammation [30, 34] (Fig. 3). Conversely, the lack of increased $T_{2}$ signal is generally associated with chronic inflammation. LGE with a normal $T_{2}$ signal can be instead indicative of resolving edema. CMR is also the preferred imaging modality to evaluate, through myocardial LGE, the presence and degree of myocardial involvement eventually associated with pericarditis and may also be used in stable patients with suspected constrictive evolution [30, 34]. CMR has, however, some limitations, which include elevated costs and relatively restricted availability, the need for a stable heart rhythm, and contraindication of gadolinium use in subjects with advanced kidney disease. Main findings at CMR are summarized in Fig. 3.

As the amount of information for a timely diagnosis of acute or recurrent pericarditis is progressively increasing, these elements must be considered as a whole for a tailored therapy according to two different phenotypes (Fig. 1).

## 4. Therapy: IL-1 Blockade as a Game Changer in Recurrent Pericarditis

Currently available agents targeting the NLRP3 inflammasome and/or IL-1 are colchicine, anakinra, and rilonacept [40, 41, 42].

### 4.1 Colchicine

The first description of colchicine in acute pericarditis dates back to 1987. Based on previous experiences in patients with recurrent polyserositis in the context of familial Mediterranean fever [43], three patients with glucocorticoid-dependent recurrent pericarditis (two idiopathic and one associated with systemic lupus erythematosus) were treated with colchicine 1 mg daily [44]. Indeed, they experienced a long recurrence-free period lasting 15 to 36 months and were able to stop glucocorticoids after two months while on a 0.5 mg daily dose [44]. In 2005, the first two randomized, open-label trials using colchicine for the treatment of acute and recurrent pericarditis were published, namely the COPE (Colchicine for acute Pericarditis) and CORE (Colchicine for Recurrent pericarditis) trials [45, 46] (Table 2, Ref. [45, 46, 47, 48, 49, 50, 51, 52, 53, 54]). Colchicine (loading dose 1 to 2 mg, maintenance 0.5 to 1 mg daily) was used together with NSAIDs for 3 to 6 months, and it was shown to reduce symptoms after 72 h as well as first and following recurrences [45, 46]. Colchicine was further investigated in other randomized, double-blind trials for patients with either acute or recurrent pericarditis (Table 2). The CORP (Colchicine for recurrent pericarditis), ICAP (Investigation on Colchicine for Acute Pericarditis), and CORP-2 trials tested weight-adjusted colchicine (0.5 mg once daily for patients <70 kg or 0.5 mg twice daily, no loading dose) for 3 months in acute pericarditis and 6 months in recurrent pericarditis confirming previous results, also in patients experiencing ≥2 recurrences [47, 48, 49]. Colchicine was found equally effective also in patients experiencing post-pericardiotomy syndrome, as shown in three randomized trials (Finkelstein *et al*. [50], COPPS [Colchicine for the Prevention of the Post-pericardiotomy Syndrome], and COPPS-2) [50, 51, 52] (Table 2).

**Table 2.** **Randomized controlled trials that tested colchicine in acute and recurrent pericarditis**.

Study	Study design	Treatment	Patients	Key results
Acute pericarditis				
COPE trial [45]	Open-label	Aspirin	120 patients (mean age 56.9 ± 18.8 years, 54 males)	Colchicine significantly decreased recurrence rate (at 18 months: 10.7% vs. 32.3% for aspirin alone, *p* = 0.004, NNT = 5) and symptom persistence at 72 hours (11.7% vs. 36.7% for aspirin alone, *p* = 0.003).
		vs.		
		aspirin + colchicine:		
		∙ loading dose: 1 mg on day 1; 0.5 mg daily (if body weight <70 kg) for 3 months;		No SAEs were observed
				Colchicine was discontinued in 5 cases (8.3%) because of diarrhea.
		∙ loading dose: 1 mg twice daily; 0.5 mg twice daily (if body weight ≥70 kg) for 3 months		
ICAP trial [48]	Double-blind	Aspirin/ibuprofen +Placebo	240 patients (mean age 52.1 ± 16.9 years, 60% males)	Colchicine reduced the occurrence of incessant or recurrent pericarditis (16.7% vs. 37.5% for placebo; RRR 0.56, 95% CI 0.30 to 0.72, *p *< 0.001; NNT = 4).
		vs.		
		aspirin/ibuprofen + colchicine:		Colchicine reduced symptom persistence at 72 hours (19.2% vs. 40.0%, *p* = 0.001) and hospitalization (5.0% vs. 14.2%, *p* = 0.02).
		∙ 0.5 mg daily if body weight ≤70 kg;		
		∙ 0.5 mg twice daily if body weight >70 kg		GI disturbance was similar in the two groups. No SAEs were reported.
Sambola *et al*. [53]	Open-label	Aspirin/NSAIDs alone	110 patients (mean age 44 ± 18.3 years, 83.6% males)	No differences in the rate of recurrences was found (13.5% vs. 7.8%, *p* = 0.34).
		vs.		
		aspirin/NSAIDs + colchicine:		
		∙ 0.5 mg twice daily if body weight <70 kg;		
		∙ 1 mg twice daily if body weight ≥70 kg		
Recurrent pericarditis				
CORE trial [46]	Open-label	Aspirin	88 patients (55.2% males)	Colchicine reduced the recurrence rate (at 18 months 24% vs. 50%, *p* = 0.02; NNT = 4) and symptom persistence at 72 hours (10% vs. 31%, *p* = 0.03).
		vs.		
		aspirin + colchicine:		
		∙ loading dose: 1 mg on day 1; 0.5 mg daily (if body weight <70 kg) for 6 months;		Colchicine allowed for a longer symptom-free interval (17.2 ± 12.2 months vs. 10.6 ± 9.6 months, *p* = 0.007).
		∙ loading dose: 1 mg twice daily; 0.5 mg twice daily (if body weight ≥70 kg) for 6 months		Diarrhea led to drug discontinuation in 7% of colchicine-treated patients. No SAEs were reported.
CORP trial [47]	Double-blind	Aspirin/ibuprofen +Placebo	120 patients (52.5 males)	Colchicine decreased the recurrence rate at 18 months (24% vs. 55%, *p *< 0.001; NNT = 3) and symptom persistence at 72 h (23% vs. 53%, *p *< 0.001) as well as remission rate at 1 week (82% vs. 48%, *p *< 0.001).
		vs.		
		aspirin/ibuprofen + colchicine:		
		∙ loading dose: 1 mg on day 1; 0.5 mg daily (if body weight <70 kg) for 6 months;		GI intolerance was the main side effect and was balanced between groups. No SAEs were observed.
		∙ loading dose: 1 mg twice daily on day 1; 0.5 mg twice daily (if body weight ≥70 kg) for 6 months		
CORP-2 trial [49]	Double-blind	Aspirin/NSAIDs + placebo	240 patients (mean age 48.7 ± 14.6 years, 50% males) with ≥2 recurrences	Colchicine reduced recurrences (21.6% vs. 42.5%; RR 0.49, 95% CI 0.24 to 0.65, *p *< 0.001; NNT = 5) and symptom persistence at 72 h (19.2% vs. 44.2%, *p *< 0.001).
		vs.		
		aspirin/NSAIDs + colchicine:		
		∙ 0.5 mg daily if body weight ≤70 kg for 6 months;		Colchicine was effective in inducing remission at 1 week (83.3% vs. 59.2%, *p *< 0.001), reducing incessant course (8.3% vs. 26.7%, *p *< 0.001) and pericarditis-related hospital admissions (1.7% vs. 10%, *p* = 0.001).
		∙ 0.5 mg twice daily if body weight >70 kg twice daily (if body weight ≥70 kg) for 6 months		
				GI intolerance was the main side effect and was similar between groups. No SAEs were observed.
Post-pericardiotomy syndrome				
Finkelstein *et al*. [50]	Open-label	On 3rd post-operative day, placebo or colchicine (0.5 mg three times daily for 1 month	111 patients (73% males)	No difference was observed for the occurrence of PPS was diagnosed between colchicine and placebo groups (10.6.% vs. 21.9%, *p *< 0.135, trend level).
COPPS [51]	Double-blind	On 3rd post-operative day,	360 patients (mean age 65.7 ± 12.3 years, 66% males)	Colchicine reduced PPS incidence at 12 months (8.9% vs. 21.1%, *p* = 0.002; NNT = 8) and the secondary endpoint including PPS-related hospitalization, cardiac tamponade, constrictive pericarditis, and relapse at 18 months (0.6% vs. 5.0%, *p* = 0.024; NNT = 22).
		Standard therapy + placebo		
		vs.		
		standard therapy + colchicine:		
		∙ loading dose: 1 mg twice daily on day 1; 0.5 mg twice daily (if body weight ≥70 kg) for 1 month;		GI disturbance occurs most frequently in the colchicine group. No SAEs were observed.
		∙ loading dose: 1 mg daily on day 1; 0.5 mg daily (if body weight <70 kg) for 1 month		
COPPS-2 [52]	Double-blind	From 48 to 72 hours before surgery, placebo	360 patients (mean age 67.5 ± 10.6 years, 68.9% men)	Colchicine decreased PPS occurrence (19.4% vs. 29.4%, *p *< 0.01; NNT = 10) but failed to reduce occurrence of AF (34% vs. 42%) or pericardial/pleural effusion 57% vs. 59%).
		vs.		
		colchicine:		
		∙ 0.5 mg twice daily (if body weight ≥70 kg) for 1 month;		
		∙ 0.5 mg daily (if body weight <70 kg) for 1 month		
Meurin *et al*. [54]	Double-blind	Placebo	197 patients (86.3% males)	Colchicine failed to reduce pericardial effusion or late cardiac tamponade (7% vs. 6%).
		vs.		
		colchicine:		Diarrhea was frequently occurred among patients on colchicine. No SAEs were recorded.
		∙ Loading dose: 1 mg twice daily; 1 mg daily (if body weight ≥70 kg) for 14 days;		
		∙ no loading dose; 1 mg daily (if body weight <70 kg) for 14 days		

Legend. AF, atrial fibrillation; CI, confidence interval; GI, gastrointestinal; NNT, number needed to treat; PPS, post-pericardiotomy syndrome; RRR, relative risk reduction; SAE, serious adverse effect.

In sum, colchicine in addition to standard anti-inflammatory therapy demonstrated to reduce recurrences up to 50% in acute and recurrent pericarditis as well as in post-pericardiotomy syndrome [55]. Over the past years, several meta-analyses supported the benefits of colchicine [56, 57, 58, 59, 60, 61, 62, 63, 64, 65, 66]. The most recent one [67], including all 6 available randomized clinical trials in acute and recurrent pericarditis (914 patients in total), demonstrated a significant lower recurrence and treatment failure rate with colchicine when compared to control (odds ratio [OR] 0.37, 95% confidence interval [CI] 0.27 to 0.51, and OR 0.29, 95% CI 0.21 to 0.41, respectively).

### 4.2 Anakinra

Nineteen case reports or series described a total of 65 patients (including both adults and children) treated with anakinra for a variable number of recurrences [68]. Among them, 77% (50/65) were treated with NSAIDs, while 92% (60/65) were both on colchicine and glucocorticoids. Recurrences occurred in 3% (2/65) of patients receiving full-dose anakinra (i.e., 100 mg once daily), 61% (21/34) experienced recurrent pericarditis following anakinra interruption, and 56% (35/62) still had recurrences after anakinra therapy was instituted [68].

In a small clinical proof-of-concept study by Wohlford *et al*. [69], five patients with acute pericarditis (three with a first episode and two with recurrent pericarditis) already treated with colchicine and NSAIDs (but not glucocorticoid-dependent) and experiencing moderate-to-severe pericarditis-related chest pain (initial or subsequent episode) were prescribed anakinra 100 mg subcutaneously within 24 hours of presentation. Anakinra significantly reduced pain, and no patients required rescue pain medication. In addition, IL-6 levels were also reduced considerably, and no treatment-related adverse events occurred [69] (Table 3, Ref. [7, 9, 69, 70]).

**Table 3.** **Clinical studies that tested IL-1 blockers in acute and recurrent pericarditis**.

Study	Study design	Treatment	Patients	Key results
Wohlford *et al. * [69]	Prospective open-label study	Anakinra 100 mg subcutaneously within 24 hours of hospital admission	6 patients with acute pericarditis with moderate-to-severe chest pain	Anakinra was administered a median of 20 h after hospital admission. Pain score decreased from a baseline of 6 (6–7.5) to 4 (2.5–4) after 6 h and to 2 (1.5–2.5) after 24 h (*p* = 0.012 and *p* = 0.002, respectively).
				IL-6 levels reduced within 24 h (95.3 [24.2–155.1] → 23.9 (4.5–71.9) pg/mL, *p* = 0.037) following anakinra administration.
				Pain reduction at 24 h was correlated with IL-6 reduction at 24 h (r = +0.966, *p* = 0.007).
				No AEs were described.
AIRTRIP study [7]	Double-blind, placebo-controlled, randomized withdrawal trial	Anakinra at 2 mg/kg daily (up to 100 mg) subcutaneously for 2 months.	21 patients (n = 11 anakinra, n = 10 placebo) with recurrent pericarditis (≥3 recurrences), increased CRP, resistant to colchicine and dependent on glucocorticoid	In the open-label phase, all patients had a complete response to anakinra at day 8 as well as CRP normalized and pain rapidly reduced. All patients were able to stop glucocorticoids within 6 weeks.
		Patients who responded (i.e., resolution of pericarditis) were randomized to anakinra or placebo for 6 months or until pericarditis recurrence		During the double-blind treatment phase, pericarditis recurrence was experienced by 90% (n = 9/10) patients in the placebo group vs. 18.2% (n = 2/11) patients in the anakinra group (incidence rate, 0.11% of patients per year).
				Median time-to-flare was 72 (64–150) days after randomization in the placebo group, whereas it could not be computed in patients randomized to anakinra (*p *< 0.001). Mean time-to-flare was 28.4 vs. 76.5 days in the placebo and anakinra groups, respectively (absolute mean difference of –48.1, 95% CI, –118.1 to 21.9 days).
				The most common AE in patients treated with anakinra was a local skin reaction at the injection site (95% patients).
IRAP study [70]	Multicenter observational cohort study	Anakinra 100 mg daily subcutaneously	224 patients with glucocorticoid-dependent and colchicine-resistant recurrent pericarditis	Recurrences occurred in 35% (n = 78/224) patients with a median flare-free of 10 months (5–18).
				After anakinra treatment, a median of zero recurrences occurred with an 83% reduction in recurrence rate (RR 0.17, 95% CI 0.14–0.20, *p *< 0.001). After 36 months from anakinra initiation, 72% patients experienced none or at most one recurrence.
				A reduction of 91% for ED admissions (RR 0.09, 95% CI 0.06–0.13, *p *< 0.001) and 86% for hospitalizations (RR 0.14, 95% CI 0.11–0.19, *p *< 0.001) was observed in patients treated with anakinra.
				During follow-up, 8.9% were admitted to the hospital for pericardiectomy and discontinued anakinra.
				After anakinra treatment, glucocorticoids were tapered and NSAIDs suspended in most patients without recurrences (27% and 24% still on glucocorticoid and NSAID therapy, respectively; 58% on colchicine).
				Transient skin reaction at the injection site was the most frequent AE (38% of patients). Arthralgias and myalgias were found in 6% of patients, while 3% experienced infections during follow-up.
RHAPSODY study [9]	Phase 3 multicenter, double-blind, event-driven, randomized-withdrawal trial	Rilonacept as a loading dose of 320 mg (or 4.4 mg/kg in patients <18 years of age) subcutaneously, followed by weekly doses of 160 mg (or 2.2 mg/kg in patients <18 years of age) subcutaneously	86 patients in the 12-week run-in period.	Rilonacept greatly lowered risk of recurrences compared to placebo (HR 0.04, 95% CI 0.01–0.18, *p *< 0.001). Median time to recurrence in the placebo group was 8.6 weeks, while it was not possible to compute this period in the rilonacept group because of too few events.
			61 patients who experienced clinical response during the run-in period (CRP ≤0.5 mg/dL and no or minimal pain while on rilonacept monotherapy without recurrences) were randomized to continue rilonacept (n = 30) or placebo (n = 31)	Injection-site skin reactions and upper respiratory tract infections were the most common AEs.

Legend. AE, adverse event; AIRTRIP, Anakinra-Treatment of Recurrent Idiopathic Pericarditis; CI, confidence interval; CRP, C-reactive protein; ED, emergency department; HR, hazard ratio; IL-6, interleukin-6; IRAP, International Registry of Anakinra for Pericarditis; NSAID, non-steroidal anti-inflammatory drug; RHAPSODY, Rilonacept inHibition of interleukin-1 Alpha and beta for recurrent Pericarditis, a pivotal Symptomatology and Outcomes stuDY; RR, rate ratio.

The first clinical trial of anakinra in recurrent pericarditis is the double-blind, placebo-controlled medication withdrawal trial AIRTRIP (Anakinra-Treatment of Recurrent Idiopathic Pericarditis) [7] (Table 3). Twenty-one colchicine-resistant, glucocorticoid-dependent patients with recurrent pericarditis and systemic inflammation were given anakinra for 60 days and then randomized to either anakinra 100 mg daily, or placebo for another 6 months. All patients had a complete response to anakinra by day 8 that persisted until randomization (day 60) [7]. Patients assigned to anakinra were able to successfully discontinue glucocorticoids within six weeks. Flares of pericarditis were significantly reduced, occurring in nine out of 10 (90%) patients in the placebo arm, and two out of 11 (18.2%) patients in the anakinra arm during the double-blind treatment withdrawal phase. After randomization, median flare-free survival was 72 days in the placebo group, whereas it could not be calculated in the anakinra group (*p *< 0.001). In patients with recurrences, the mean time to flare was 28.4 days vs. 76.5 days in the placebo and anakinra groups, respectively [7]. Localized skin reactions at the site of injection were the most common adverse effect.

The IRAP (International Registry of Anakinra for Pericarditis) is a registry of 224 colchicine-resistant, glucocorticoid-dependent patients (46 ± 14 years old, 63% women, 75% idiopathic) with recurrent pericarditis and elevated CRP levels receiving colchicine and NSAIDs, who were treated with anakinra [70] (Table 3). Following anakinra treatment, a median of zero recurrences was observed, with an 83% reduction in recurrence rate and a mean of 1 recurrence every 939 days. Similarly, after 36 months of anakinra treatment, almost three-quarters of patients experienced 0 to 1 recurrence. During follow-up, no need for emergency department visits nor hospitalizations was recorded, with a nearly 90% reduction compared with the period before anakinra treatment. Regarding glucocorticoids, treatment with anakinra allowed to successfully taper and suspend these drugs, with <30% still on glucocorticoid therapy. No serious adverse events were recorded, while injection site reactions occurred in 38% of the patients. Six patients experienced infections that resolved with appropriate treatment, with half of them needing temporary interruption of anakinra.

### 4.3 Rilonacept

First data with rilonacept in recurrent pericarditis derive from the phase II, multicenter, single-arm, open-label clinical trial RHAPSODY. In this study, either patients with at least a second recurrence and glucocorticoid-dependent recurrent pericarditis (no active recurrence, but at least two previous episodes) received subcutaneous rilonacept 320 mg (loading dose) with 160 mg weekly (maintenance dose) for 5 additional doses followed by an optional 18-week on-treatment extension period (option to wean background therapy) when already receiving conventional therapies (colchicine, NSAIDs, glucocorticoids) [71]. In symptomatic patients, chest pain was reduced, and CRP rapidly normalized in all patients. In addition, prednisone was successfully discontinued in 11 out of 13 patients (84.6%), with no patient experiencing recurrent pericarditis during this time [71]. Notably, the number of pericarditis episodes per year was nearly zero. The positive results of this phase II study found rilonacept to be safe, with most of the adverse events being mild-to-moderate in severity, primarily injection site reactions [71]. Among additional endpoints, a general improvement in the health-related quality of life was seen in symptomatic patients with increased CRP levels [72], while the exploratory cardiac magnetic resonance imaging substudy (11 patients) showed improvement in pericardial inflammation [73].

These promising results were confirmed in the phase III RHAPSODY study, a double-blinded, placebo-controlled, multicenter randomized-withdrawal trial in colchicine-resistant, glucocorticoid-dependent patients with symptomatic recurrent pericarditis [9] (Table 3). After an initial 12-week run-in period, during which all patients received rilonacept as a subcutaneous injection (loading dose 320 mg followed by 160 mg weekly thereafter), patients who responded favorably to rilonacept monotherapy (in terms of improvement in CRP and chest pain) were eligible to enter the randomized-withdrawal period, where they were randomly assigned in a 1:1 ratio to continue rilonacept or a matching dose of placebo each week. During the run-in phase, a quick and persistent improvement of chest pain and systemic inflammation was observed, with a median time to pain response of 5 days, and a median time to CRP normalization of 7 days. The median time required for patients to discontinue background therapy and continue with rilonacept monotherapy was 7.9 weeks. Of note, all patients on glucocorticoids were able to stop them and started receiving rilonacept monotherapy during the run-in period. A recent *post-hoc* analysis has shown that the transition from background therapies to rilonacept monotherapy occurred without recurrences, irrespective of a sequential or concurrent tapering approach [74]. Regarding the randomized-withdrawal period, rilonacept strikingly decreased the risk of pericarditis recurrence compared with placebo (hazard ratio 0.04, 95% confidence interval 0.01 to 0.18, *p *< 0.001). During this phase, two out of 30 patients (7%) in the rilonacept group vs. twenty-three out of 31 patients (74%) in the placebo group experienced a pericarditis recurrence event. Importantly, recurrences in the rilonacept group were motivated by temporary interruptions of the trial-drug regimen. By taking a look to the long-term extension study, only one recurrence in subjects on rilonacept (associated with a 4-week interruption) compared with 75% recurrence rate (n = 6/8) in the off-treatment observation group was recorded (hazard ratio 0.018, *p *< 0.0001) [75]. Major secondary efficacy endpoints (assessed at week 16 of the randomized-withdrawal period) highlighted the positive effect of rilonacept vs. placebo on persistent clinical response (81% vs. 20%, *p *< 0.001) and improvement of pericarditis symptoms (81% vs. 25%, *p *< 0.001). Importantly, no patient in the randomized-withdrawal period had to re-introduce glucocorticoid therapy. Improvements in patient-reported quality of life, symptom severity, pain and sleep, while on rilonacept were recorded [76]. As demonstrated in the previous phase II trial, injection-site reactions and infections (especially of the upper respiratory tract) were the most common adverse events, with only 5 serious adverse reactions and no death during the whole trial.

In sum, the rapid resolution of pain (median five days), CRP normalization (median time of seven days), effective withdrawal of glucocorticoids, and the lack of recurrences in the treatment group following a randomized-withdrawal period provide confirmatory evidence that rilonacept monotherapy is sufficient to maintain disease control [42]. Hence, rilonacept is not only able to provide a rapid resolution of the acute flare of pericarditis but warrants successful maintenance of remission during rilonacept monotherapy. As of March 2021, rilonacept was approved by the FDA for the treatment of recurrent pericarditis [10] following the results of the RHAPSODY trial [9].

### 4.4 Canakinumab

Evidence on the benefits of canakinumab in recurrent pericarditis is unclear, and described only in case reports and relatively small case series [77, 78, 79, 80]. Moreover, recurrences have been described after switching from anakinra to canakinumab in patients with good response to anakinra [81].

### 4.5 The Importance of Phenotyping Patients with Recurrent Pericarditis

**Fig. 4.**
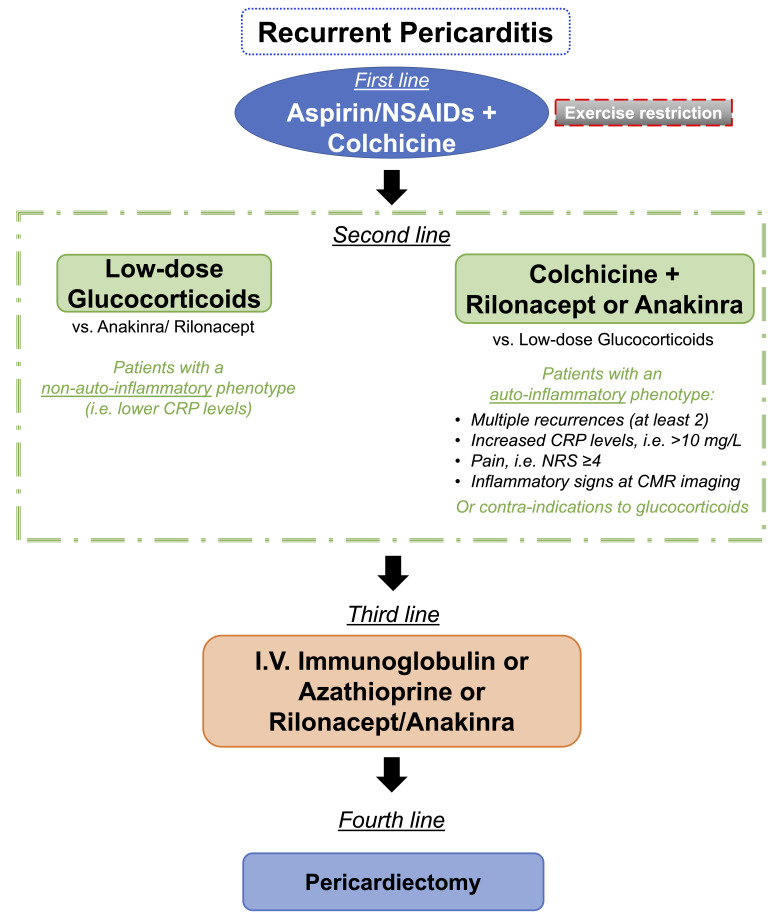
**Suggested flowchart for the use of IL-1 inhibitors in patients with recurrent pericarditis**. In patients experiencing ≥2 recurrences, it is important to evaluate specific disease phenotypes to tailor the therapeutic strategy. For those with an auto-inflammatory phenotype, an IL-1 inhibitor should be the drug of choice compared with glucocorticoids. On the contrary, for patients presenting without a clear auto-inflammatory phenotype, low-dose glucocorticoids could be evaluated along with IL-1 inhibitors on a case-by-case basis.

A subset of patients experiences multiple recurrences (≥2 recurrences). When first-line treatments fail, it is of utmost importance to phenotype patients to offer them tailored therapies [82]. Patients with increased levels of CRP and multiple recurrences are more likely to benefit from IL-1 blockade. In this case, an auto-inflammatory mechanism is generally believed to be the cause of the acute flare. Along with inflammatory biomarkers, these patients should undergo routine CMR in the diagnostic work-up to promptly assess pericardial inflammation, which might be an important prognostic factor. In patients with an auto-inflammatory mechanism of disease, pharmacological blockade of IL-1 can blunt inflammation and help resolve pericardial inflammation and control symptoms. In this context, colchicine must not be discontinued while starting IL-1 blockers to synergize the inhibition of the NLRP3 inflammasome/IL-1β axis. In patients with recurrent pericarditis without a frank increase in inflammatory biomarkers (e.g., CRP), low-dose glucocorticoids might be the treatment of choice since auto-inflammation is less likely to be the primary driver of the acute flare. However, additional signs (e.g., evidence of pericardial inflammation at CMR) should be considered before starting an IL-1 blocker. These concepts are summarized in Fig. 4 and have been recently discussed elsewhere [82].

## 5. Conclusions

In the past years, substantial advances in the understanding of acute and recurrent pericarditis have been accomplished. As an etiologic diagnosis is often unfeasible or fails, in most cases, acute pericarditis has been regarded to as “idiopathic”. This has probably prevented the medical community for many years from more in-depth mechanistic research aimed at pathophysiology and targeted therapies. Thanks to clinical and pre-clinical studies conducted in recent times, it is now clear that acute pericarditis is an inflammatory condition that can be triggered by infectious or non-infectious stimuli, except for those cases due to an autoimmune disease (e.g., systemic lupus erythematosus, rheumatoid arthritis). In most cases, the acute inflammation of the pericardium completely resolves. Almost 30% of patients may, however, experience recurrences, that result from a rapid tapering of anti-inflammatory drugs or alternatively from a not adequately controlled autoinflammatory phenomenon. The latter is likely to depend on a sustained production of IL-1β that stimulates the additional release of IL-1α and IL-1β, thus fueling the vicious circle of pericardial inflammation [83]. As a further proof, patients not treated with colchicine during the first episode are at higher risk of recurrence [84]. On the contrary, pharmacological agents targeting IL-1 — anakinra and rilonacept — greatly reduced recurrence rates [7, 9, 85]. Recently, Peet *et al*. [29] have shown that idiopathic recurrent pericarditis is associated with *MEFV* gene variants, that are involved in IL-1β overactivity in Mediterranean fever, a prototypical systemic autoinflammatory disease presenting with recurrent serositis. These findings collectively support the role of the NLRP3 inflammasome/IL-1β axis as a pivotal mediator in the pathophysiology of recurrent pericarditis, and corroborate the use of targeted therapies to block the inflammasome [6].

An additional important point deals with patients’ phenotyping in order to provide a tailored therapy. Recent studies identified a central role for CMR in the diagnosis of acute pericarditis (e.g., LGE sequence and edema-weighted T2-weighted short-tau inversion recovery sequence) in patients experiencing multiple recurrences [34, 38, 86]. CMR can be coupled with the measurement of inflammatory biomarkers to recognize patients at higher risk for complications [87, 88]. This allows a more cautious tapering of anti-inflammatory therapies which should take place when both inflammatory biomarkers are lowering or negative, and pericardial LGE has resolved [37, 39].

Given the increased pathophysiological understanding of acute and recurrent pericarditis and recent solid evidence from clinical studies, it is time for guidelines to incorporate novel treatments targeting the NLRP3 inflammasome/IL-1β axis. In the next future, research should be focused on the selective pharmacological blockade of the NLRP3 inflammasome to address additional pathogenetic mechanisms involved in acute and recurrent pericarditis.
